# Appearance of fluid content in Rathke’s cleft cyst is associated with clinical features and postoperative recurrence rates

**DOI:** 10.1007/s11102-024-01395-y

**Published:** 2024-05-18

**Authors:** Takamitsu Iwata, Satoru Oshino, Youichi Saitoh, Manabu Kinoshita, Yuji Onoda, Noriyuki Kijima, Kosuke Mukai, Michio Otsuki, Haruhiko Kishima

**Affiliations:** 1 Department of Neurosurgery, Osaka University Graduate School of Medicine, 2-2 Yamadaoka, Suita, Osaka 567-0872 Japan; 2 Department of Mechanical Science and Bioengineering, Osaka University Graduate School of Engineering Science, Toyonaka, Osaka Japan; 3 Tokuyukai Rehabilitation Clinic, Toyonaka, Osaka Japan; 4 Department of Neurosurgery, Asahikawa Medical University, Asahikawa, Hokkaido Japan; 5 Department of Metabolic Medicine, Osaka University Graduate School of Medicine, Suita, Osaka Japan; 6 Department of Endocrinology, Tokyo Women’s Medical University, Shinjuku-ku, Tokyo, Japan

**Keywords:** Rathke’s cleft cysts, Transsphenoidal surgery, Cyst contents, Prognosis

## Abstract

**Purpose:**

The contents of Rathke’s cleft cysts (RCCs) vary from clear and slightly viscous to purulent. Surgical treatment of symptomatic RCCs involves removing the cyst contents, whereas additional cyst-wall opening to prevent reaccumulation is at the surgeon’s discretion. The macroscopic findings of the cyst content can reflect the nature of RCCs and would aid in surgical method selection.

**Methods:**

We retrospectively reviewed the records of 42 patients with symptomatic RCCs who underwent transsphenoidal surgery at our institute between January 2010 and March 2022. According to the intraoperative findings, cyst contents were classified into type A (purulent), type B (turbid white with mixed semisolids), or type C (clear and slightly viscous). Clinical and imaging findings and early recurrence rate (within two years) were compared according to the cyst content type.

**Results:**

There were 42 patients classified into three types. Patients with type C were the oldest (65.4 ± 10.4 years), and type A included more females (92.9%). For magnetic resonance imaging, type-A patients showed contrast-enhanced cyst wall (92.9%), type-B patients had intracystic nodules (57.1%), and all type-C patients showed low T1 and high T2 intensities with larger cyst volumes. Fewer asymptomatic patients had type C. Preoperative pituitary dysfunction was most common in type A (71.4%). Early recurrence was observed in types A and C, which were considered candidates for cyst-wall opening.

**Conclusion:**

The clinical characteristics and surgical prognosis of RCCs depend on the nature of their contents.

**Supplementary Information:**

The online version contains supplementary material available at 10.1007/s11102-024-01395-y.

## Introduction

Rathke’s cleft cysts (RCCs) are benign sellar and suprasellar cystic lesions arising from epithelial remnants of Rathke’s pouch [1]. They become clinically apparent if they are large enough to compress adjacent structures or rupture, and the most common presenting symptoms include headaches, visual disturbances, and pituitary hormone abnormalities [2].

For symptomatic RCCs, transsphenoidal surgery (TSS) is usually indicated as the first-choice treatment. Surgical techniques include either removing the contents and irrigating the cyst, or a more aggressive approach, opening the cyst wall and creating a communication with the cerebral cistern to prevent content reaccumulation [3]. The former approach may increase the likelihood of recurrence, whereas the latter poses a risk for cerebrospinal fluid (CSF) leakage and, in more severe cases, may lead to pituitary dysfunction [2–4].

We believe that the surgical management of RCCs should be planned based on their pathophysiology. Prior studies have identified squamous metaplasia, suprasellar location, lesion enhancement on magnetic resonance imaging (MRI), and isointensity on T2-weighted imaging (T2WI) as risk factors for postoperative recurrence [3–5]. However, as for squamous metaplasia, it is not always possible to acquire enough surgical specimens for histological diagnosis and those findings are confirmed only after surgery.

Cyst contents exhibit a spectrum of characteristics, ranging from limpid and slightly viscous to purulent, and can be easily assessed during TSS. In this study, we investigated the intraoperative macroscopic characteristics of cyst contents under the hypothesis that they may reflect the nature and prognosis of RCCs and aid in surgical method selection.

## Methods

We retrospectively reviewed the records of patients with primary symptomatic RCCs who underwent TSS at our institute between January 2010 and March 2022. RCCs were diagnosed based on MRI findings of a cyst located around the sella turcica without mass component and the official report by the radiologist. Cases of xanthogranuloma, craniopharyngioma, and epidermoid cyst were differentiated by radiological findings and diagnoses were confirmed by histopathology findings. In cases where all surgeries during the study period were reoperations, the initial medical record was also referenced.

The cyst contents were classified into three types: type A (purulent), type B (turbid white with mixed semisolids), or type C (clear and slightly viscous) by three observers (S.O., N.K., and T.I.) who reviewed operative videos independently in a blinded manner. The classification was adopted if at least two observers selected the same type.

The following demographic and clinical data were collected and compared according to the cyst content characteristics: age, sex, MRI findings, pituitary dysfunction, surgical technique, and early recurrence. Evaluated MRI findings included RCC signal intensity on T1-weighted imaging (T1WI) and T2WI, cyst volume, presence of intracystic nodules, and cyst-wall enhancement. RCC signal intensity was determined by comparison with the intensity of the temporalis muscle under the same conditions. Cyst volumes were determined using the following formula: height × width × length / 2 and expressed in cubic millimeters.

Pituitary function was evaluated comprehensively by endocrinologists according to the Japan Endocrine Society guideline [6] with various stimulating tests depending on the patient condition. In this study, we focused on adrenal insufficiency (AI) and diabetes insipidus (DI), which were examined with priority in perioperative management. We considered patients who received hormone replacement for AI or DI by endocrinologists as having pituitary dysfunction.

Surgical procedures were classified as cyst content evacuation only or that with additional cyst-wall opening to create communication with the cistern. For opening, the cyst wall was penetrated and the hole was enlarged with forceps after careful observation of running vessels beyond the wall. Then, the deflected flap was torn off using forceps or cut with scissors depending on the situation. Cases requiring reoperation within two years after surgery due to cyst reaccumulation were defined as early recurrence cases.

As type C could be easily distinguished from other types by MRI, we collected clinical data of asymptomatic patients with asymptomatic RCCs of 60 mm^3^ or larger who were referred to our institution from 2011 to 2019 and investigated the proportion of type C.

Statistical analysis was performed using JMP Pro 16.0.0 (SAS Institute, Cary, NC). Continuous variables were presented as arithmetic means with standard deviations, while categorical variables were presented as frequencies and percentages. The Kruskal–Wallis and Wilcoxon rank-sum tests were employed to compare continuous variables and the chi-squared test to compare categorical variables. The significance level was set at 0.05.

The protocol of this study was approved by the Ethics Committee of the Osaka University Hospital (approval number 19,370). The study was conducted in accordance with the tenets of the Declaration of Helsinki. The requirement for obtaining written informed consent from each patient was waived due to the retrospective nature of the study.

## Results

The clinical characteristics according to the RCC type in 42 patients (30 females; mean age at surgery, 53.0 ± 18.0 years) are summarized in Table 1 (see Supplementary Table 1 for detailed information). Sixteen patients had AI and three had DI, preoperatively. For other pituitary axes, hypogonadism was identified in 10 patients, hypothyroidism in 11, and growth hormone deficiency in 19. Hyperprolactinemia was identified in 17, which resolved within one year after surgery except for one patient. The mean postoperative period was 3.9 ± 2.3 years and four required reoperation for early recurrence. Twenty patients (47.6%) were histologically diagnosed with RCCs. Cyst-wall opening was consistently associated with an intraoperative CSF leak; however, no patient required repair procedures for postoperative CSF leakage. As for postoperative pituitary dysfunction, 17 patients had AI (11 in type A, three in type B, three in type C) and eight patients had DI (five in type A, two in type B, one in type C).

**Table 1:** Characteristics of patients with symptomatic Rathke’s cleft cysts

		Type A (*n* = 14)	Type B (*n* = 14)	Type C (*n* = 14)	*p*-value
Sex	Female	13 (92.9%)	9(64.3%)	8 (57.1%)	0.086
	Male	1 (7.1%)	5 (35.7%)	6 (42.9%)	
Age (years)	Mean ± SD	47.7 ± 17.3	44.6 ± 18.2	65.4 ± 10.4	< 0.01
MRI T1	Low	3 (21.4%)	2 (14.3%)	14 (100.0%)	< 0.01
	Iso	4 (28.6%)	7 (50.0%)	0 (0.0%)	
	High	7 (50.0%)	5 (35.7%)	0 (0.0%)	
MRI T2	Low	6 (42.9%)	3 (21.4%)	0 (0.0%)	0.023
	Iso	2 (14.3%)	2 (14.3%)	0 (0.0%)	
	High	6 (42.9%)	9 (64.3%)	14 (100.0%)	
	Wall enhancement	13 (92.9%)	8 (57.1%)	2 (14.3%)	< 0.01
	Nodules	3 (21.4%)	8 (57.1%)	1 (7.1%)	0.01
Cyst volume (mm^3^)	Mean ± SD	2,320.4 ± 2339.6	2,385.9 ± 3105.8	3,321.7 ± 4,443.9	0.39
Symptom	Headache	4 (28.6%)	6 (42.9%)	2 (14.3%)	0.13
	Preoperative pituitary dysfunction (for AI and/or DI)	10 (71.4%)	3 (21.4%)	3 (21.4%)	0.01
	Visual disturbance	13 (92.9%)	9 (64.3%)	12 (85.7%)	< 0.01
Histological diagnosis		5 (35.7%)	7 (50.0)	8 (57.1%)	

SD, standard deviation

AI, adrenal insufficiency

DI, diabetes insipidus

### Type-A RCCs

Type-A RCCs were found in 14 patients, of whom 13 (92.9%) were women, accounting for the highest proportion among the three types (*p* = 0.086, chi-squared test). The rate of preoperative pituitary dysfunction was the highest in type A among the three types (Table 1, *p* = 0.0071 [vs. types B and C], *p* = 0.0041 [vs. type B], *p* = 0.0041 [vs. type C], chi-squared test).

**Fig. 1:**
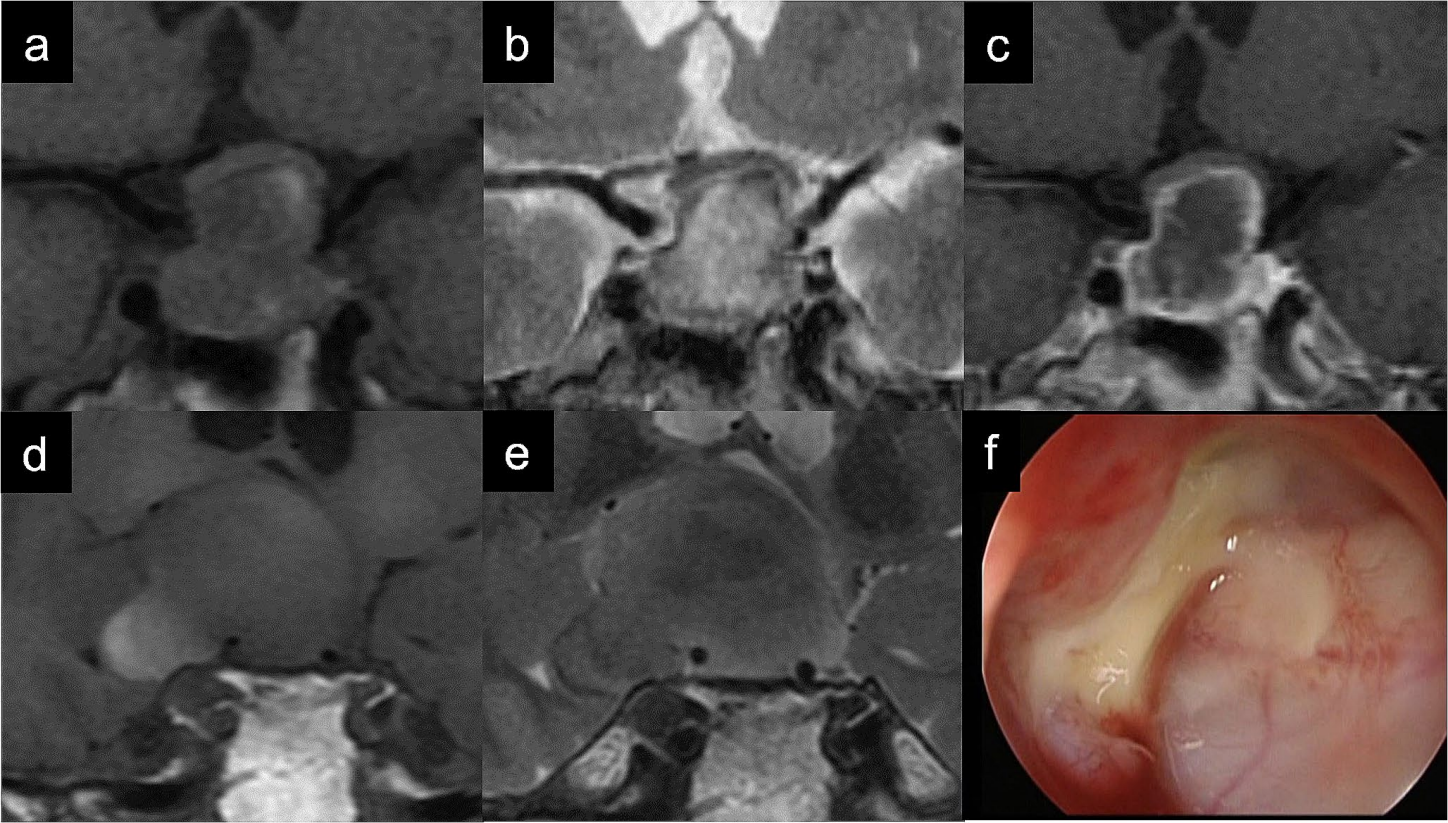
Representative recurrent case for type A Rathke’s cleft cyst (Patient No. 1). (**a**) T1-weighted image (T1WI) and (**b**) T2-weighted image (T2WI) showing mixed low and high signal intensity in the cyst; (**c**) Contrast-enhanced T1WI showing cyst wall enhancement; (**d**) T1WI obtained at the time of cyst recurrence showing high signal intensity in the lateral part of the cyst; (**e**) T2WI obtained at the time of recurrence showing mixed low and high signal intensity in the cyst; (**f**) Intraoperative findings at recurrence showing purulent contents on the lateral side of the cyst

For MRI findings, cyst-wall enhancement was observed in 13 cases (92.9%), which was significantly more frequent than that for type C (*p* < 0.01, chi-squared test). Figure 1 shows the imaging and intraoperative findings in a representative type-A case.

Because of the purulent appearance of the cyst contents, bacterial cultures were performed in 11 cases, six of which were positive. To avoid secondary infection such as meningitis, we initially had not opened the wall for type A. However, we experienced three cases with early recurrence: each had a larger cyst with suprasellar extension and, for one patient, the cyst wall was not opened because purulent content remained at the margin of the cyst (Fig. 1D-F). In fact, the patient underwent five TSS procedures within 1–2 years interval due to reaccumulation from the remnant content. This patient was cured by cyst-wall opening after completely evacuating the cyst contents with curved aspiration and extensive irrigation, and there was no recurrence for more than 3 years. The same strategy was applied in two other recurrent cases, resulting in no further recurrence. In total, three patients underwent cyst-wall opening and 11 patients did not, with no significant differences in early recurrence rates by surgical technique in type A (Table 2).

**Table 2:** Surgical technique and prognosis of symptomatic Rathke’s cleft cysts

	Type A (*n* = 14)		Type B (*n* = 14)		Type C (*n* = 14)	
	Early recurrence		Early recurrence		Early recurrence	
	+	-	+	-	+	-
Only cyst content removal	2 (14.3%)	9 (64.3%)	0 (0.0%)	6 (43.8%)	1 (7.1%)	0 (0.0%)
Cyst wall opening	1 (7.1%)	2 (14.3%)	0 (0.0%)	8 (56.2%)	0 (0.0%)	13 (92.9%)
Postoperative observation period (days)	1147.3 ± 882.1		1502.8 ± 656.7		1660.7 ± 886.3	

### Type-B RCCs

**Fig. 2:**
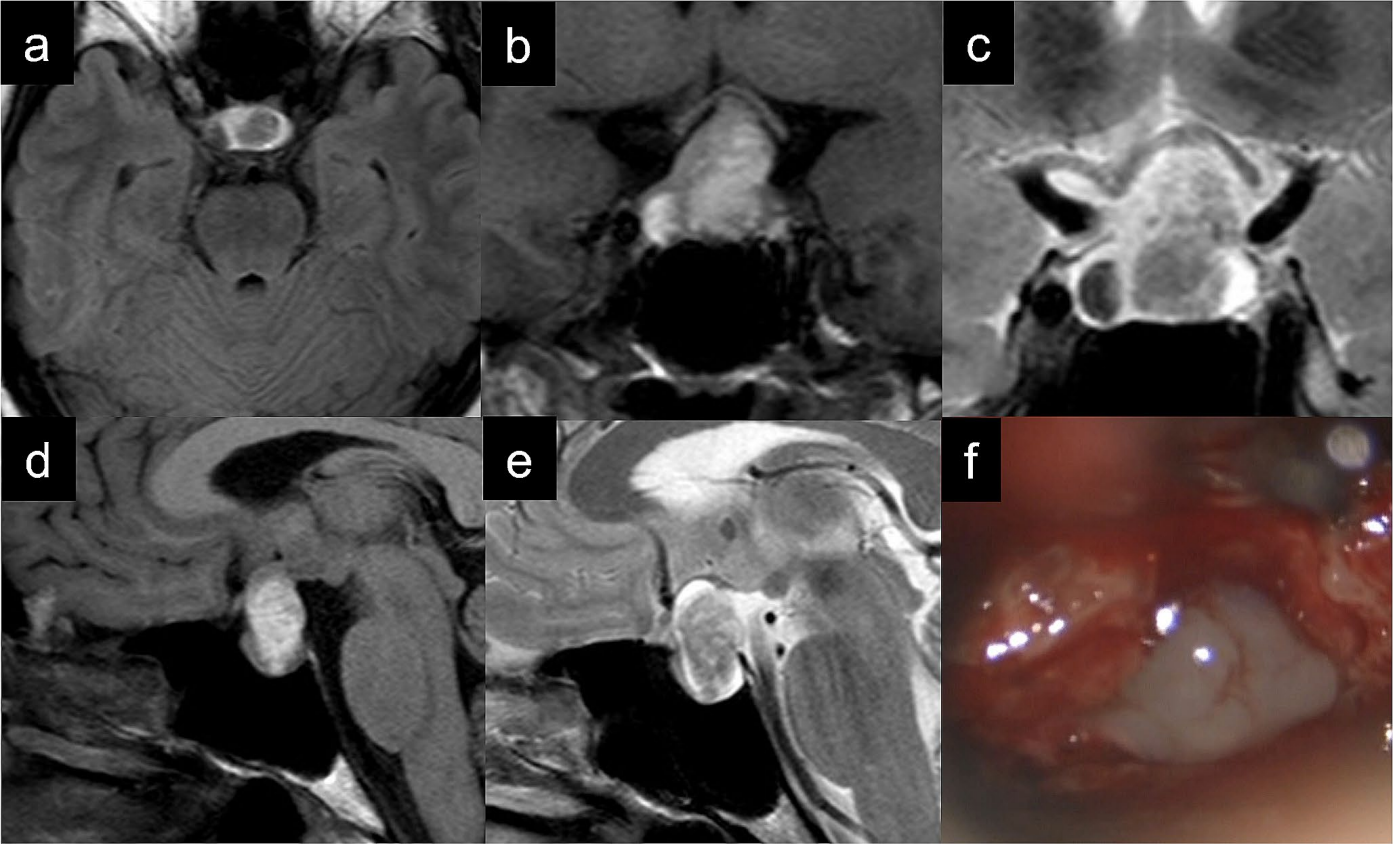
Representative case for type B Rathke’s cleft cyst (Patient No. 22). (**a**) Axial T1-weighted image (T1WI) showing iso or high intensity in the cyst; (**b**) Coronal T1WI showing nodule in the cyst; (**c**) Coronal T2-weighted image (T2WI) showing iso to low intensity in the cyst; (**d**) Sagittal T1WI showing compression of the optic nerve by the cyst; (**e**) Sagittal T2WI showing compression of the optic nerve by the cyst; (**f**) Intraoperative high-magnified image under a microscope showing that the content of the opened cyst was a clear, low-viscosity liquid with a slight whitish tinge

Type-B RCCs were observed in 14 patients. Figure 2 shows the imaging and intraoperative findings in a representative type-B case. Intracystic nodules were detected in eight patients (57.1%), which was the highest rate among the three types (*p* = 0.011 [vs. types B and C], *p* = 0.0031 [vs. type A], *p* = 0.015 [vs. type C], chi-squared test). Signal intensity on MRI differed across cases and cyst-wall enhancement was observed in eight cases (57.1%). Cyst-wall opening was performed in eight cases (57.1%), and no cases required reoperation within 2 years after surgery.

### Type-C RCCs

Type-C RCCs were found in 14 patients with a mean age of 65.4 ± 10.4 years, which was significantly older than that of the other groups (*p* = 0.0033, Kruskal–Wallis; *p* = 0.0054 [vs. type A], *p* = 0.0038 [vs. type B], Wilcoxon rank-sum test).

The cysts showed T1 low and T2 high intensity on MRI and could be reliably distinguished from the other types. As shown in Table 1, the mean cyst volume was larger and the rate of cyst-wall enhancement was significantly less than those in the other groups (*p* < 0.01[vs. types B and C], *p* < 0.0001 [vs. type A], *p* = 0.00026 [vs. type B], chi-squared test).

**Fig. 3:**
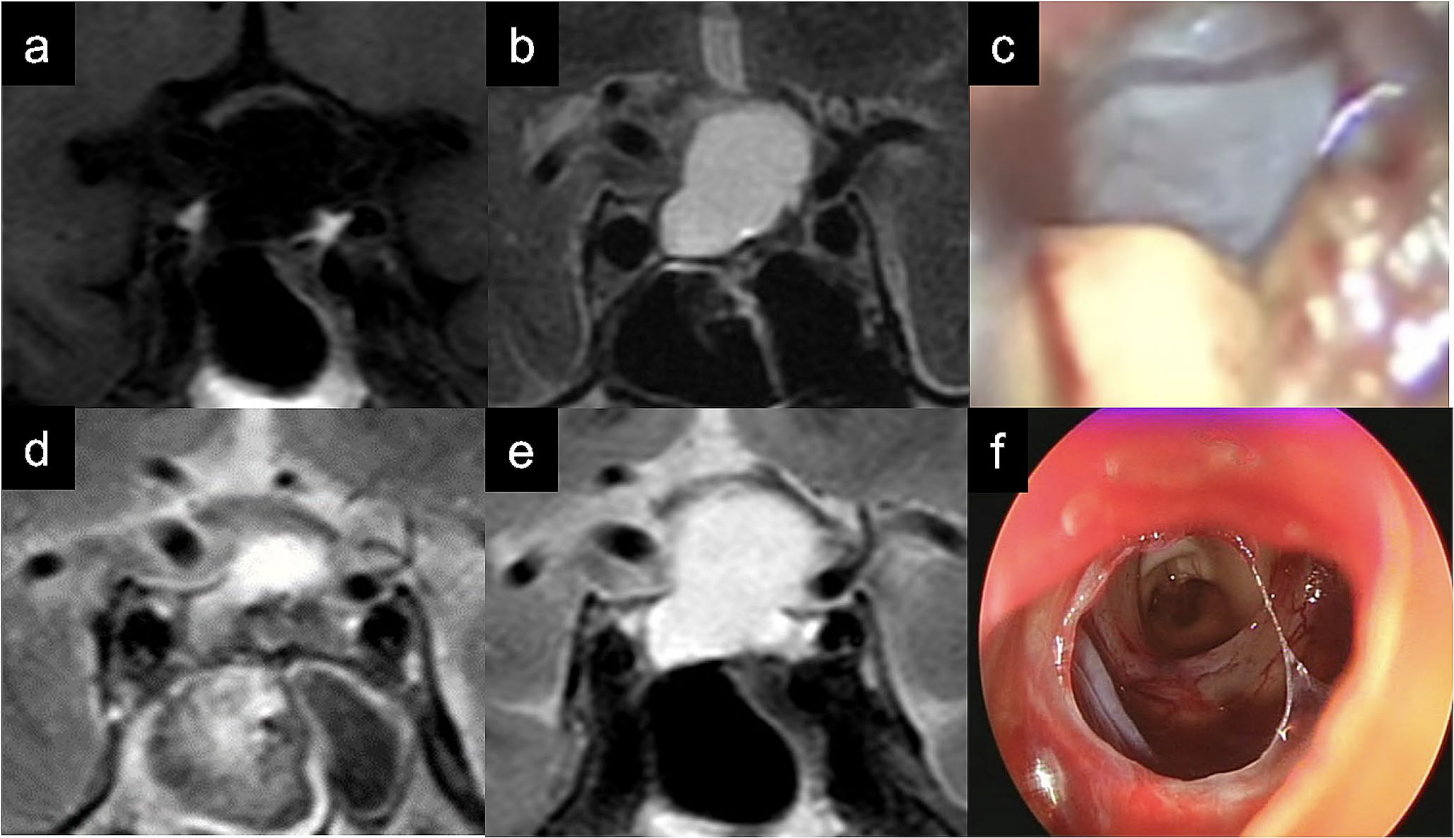
Representative case for type C Rathke’s cleft cyst (Patient No. 32). (**a**) T1-weighted image (T1WI) showing low intensity in the cyst; (**b**) T2-weighted image (T2WI) showing high intensity in the cyst; (**c**) Intraoperative findings showing the thin cyst wall, and when it was cut, clear, watery content flowed out; (**d**) T2WI obtained 1 week postoperatively showing that the cyst is shrinking; (**e**) T2WI obtained 3 months postoperatively showing that the cyst had reaccumulated and the optic nerve is compressed; (**f**) Intraoperative findings at the time of cyst recurrence showing that the cyst and cyst wall were opened and the optic nerve is observed under endoscope

Early recurrence was observed in a patient in whom the cyst wall was not opened at the initial surgery due to old age (Fig. 3). After this experience, the cyst wall was opened in all patients with this type of RCC. There was no recurrence within 2 years after the first operation, although the content was slightly reaccumulated with a change in the MRI intensity in one case.

### Asymptomatic RCCs

The clinical data of 74 patients with asymptomatic RCCs (53 females, mean age 45.2 ± 19.0 years) were available. Type C cysts were detected in nine patients (12.2%) with asymptomatic RCCs, which was significantly fewer than in the symptomatic group (14/42, 33.3%; *p* = 0.006, chi-squared test).

## Discussion

In this study, we aimed to classify RCCs into three types based on the macroscopic characteristics of the cyst contents, which are easily detected during surgery.

Firstly, we found that the clinical characteristics of RCCs could be distinguished by their contents. Type-A RCCs predominantly affected female patients and had a strong association with infection and inflammation resulting in higher portion of contrast-enhanced cyst walls and preoperative pituitary dysfunction [7, 8]. Bacteria were present in the cyst content in more than half of the examined cases; however, we could not conclude whether this proportion is high because of the lack of data on other types. Type-B RCCs demonstrated milder inflammatory manifestations compared to type A. Type-C RCCs were found primarily in older patients and showed relatively larger volumes, which was consistent with serous-type RCCs as classified by Ozoner et al. [8]. The paucity of asymptomatic type-C cysts in our study suggests a brief asymptomatic phase and rapid expansion towards symptomatic presentation in this type.

Our second finding underscores the differences in postoperative early recurrence rates according to RCC type and surgical strategy. Generally, postsurgical recurrence of RCCs has been reported in approximately 10% of cases [9–11]. Similar to previous reports [11, 12], the removal of cyst contents alone versus additional cyst-wall opening did not show a difference in the early recurrence rate in type-A and -B cysts. In contrast, our data suggested that cyst-wall opening will reduce the recurrence rate in type C, in which the thin and translucent walls can be opened safely. In addition, cyst-wall opening after removal of all content will be effective for large type-A cysts, which tend to recur when purulent fluid remains.

Kino et al. [13] proposed a method in which the RCC epithelium and the mucosa of the sphenoid sinus are connected to maintain persistent drainage and prevent recurrence. In addition to surgical techniques, our study emphasizes the importance of adopting surgical strategies tailored according to the RCC type based on the intraoperative findings to optimize patient outcomes.

This study has some limitations. First, it was a single-center retrospective study with a relatively small sample size. Cyst content assessment was subjective although it was performed in a blinded manner. The location and size of cyst-wall opening were not consistent because they depended on the intraoperative situation. In addition, cyst-wall opening was performed predominantly in recurrent cases or with hypopituitarism. Therefore, it would be inappropriate to state that cyst-wall opening relates to pituitary dysfunction from this study. Histological diagnosis was not confirmed in half of the patients in our study because of the difficulty of obtaining enough specimens during surgery. However, we consider it possible to clinically diagnose the majority of RCCs by imaging and intraoperative findings of cyst content. Future research including pathological findings and inflammatory markers should be conducted for a more objective analysis.

## Conclusion

The macroscopic characteristics of the cyst content were classified into three types and each type reflected the nature of RCCs. Our classification could be an aid for neurosurgeons to select strategies during surgery for RCCs.

### Electronic supplementary material

Below is the link to the electronic supplementary material.

Supplementary Material 1
