# Supplementary material for: Appearance of fluid content in Rathke’s cleft cyst is associated with clinical features and postoperative recurrence rates
Source: Pituitary. 2024 May 18;27(3):287–93. doi: 10.1007/s11102-024-01395-y (PMC11150284; doi:10.1007/s11102-024-01395-y)
Supplement: Supplementary file 1 — Supplementary Material 1 [file 11102_2024_1395_MOESM1_ESM.docx]

| Case No. | Group | Sex | Age | Pre-operative  Corticosteroid replacement | Pre-operative antidiuretic hormone replacement | Post-operative corticosteroid replacement | Post-operative antidiuretic hormone replacement | Cyst volume (mm^3^) | Cyst wall opening | Early recurrence |
| --- | --- | --- | --- | --- | --- | --- | --- | --- | --- | --- |
| 1 | A | F | 52 | Yes | No | Yes | No | 10000 | No | Yes |
| 2 | A | F | 46 | No | No | No | Yes | 1941 | No | No |
| 3 | A | F | 65 | No | No | Yes | No | 1063 | No | No |
| 4 | A | F | 23 | Yes | Yes | Yes | Yes | 2059 | No | No |
| 5 | A | F | 43 | Yes | No | No | Yes | 2006 | No | No |
| 6 | A | F | 47 | Yes | No | Yes | No | 370 | No | No |
| 7 | A | F | 44 | No | No | Yes | No | 1163 | No | Yes |
| 8 | A | F | 23 | Yes | No | Yes | Yes | 2656 | No | No |
| 9 | A | F | 69 | Yes | No | Yes | No | 505 | No | No |
| 10 | A | F | 47 | Yes | No | Yes | No | 2145 | No | No |
| 11 | A | F | 44 | No | No | Yes | No | 1853 | Yes | No |
| 12 | A | F | 24 | Yes | Yes | Yes | No | 2398 | Yes | No |
| 13 | A | F | 59 | Yes | No | No | Yes | 1315 | Yes | Yes |
| 14 | A | M | 82 | Yes | No | Yes | No | 3005 | No | No |
| 15 | B | M | 17 | Yes | No | No | No | 2305 | Yes | No |
| 16 | B | F | 29 | No | No | No | No | 685 | No | No |
| 17 | B | M | 64 | No | No | No | No | 1242 | No | No |
| 18 | B | F | 17 | No | No | No | No | 1252 | Yes | No |
| 19 | B | F | 45 | No | No | No | No | 1494 | Yes | No |
| 20 | B | F | 37 | No | No | No | No | 869 | Yes | No |
| 21 | B | M | 38 | Yes | No | Yes | No | 2026 | Yes | No |
| 22 | B | F | 41 | No | No | No | No | 2633 | Yes | No |
| 23 | B | F | 68 | No | No | Yes | No | 4007 | Yes | No |
| 24 | B | M | 78 | No | No | No | No | 926 | No | No |
| 25 | B | F | 58 | No | No | No | No | 1429 | No | No |
| 26 | B | F | 34 | No | No | No | No | 2797 | No | No |
| 27 | B | M | 41 | No | No | No | No | 1584 | No | No |
| 28 | B | F | 64 | Yes | Yes | Yes | Yes | 1216 | Yes | No |
| 29 | C | M | 54 | No | No | No | No | 1350 | Yes | No |
| 30 | C | F | 45 | No | No | No | No | 18098 | Yes | No |
| 31 | C | M | 67 | Yes | No | Yes | No | 2958 | Yes | No |
| 32 | C | F | 79 | No | No | No | No | 1484 | No | Yes |
| 33 | C | F | 76 | No | No | No | No | 1344 | Yes | No |
| 34 | C | M | 75 | Yes | No | Yes | No | 1283 | Yes | No |
| 35 | C | F | 57 | No | No | No | No | 1927 | Yes | No |
| 36 | C | F | 72 | No | No | No | No | 2634 | Yes | No |
| 37 | C | F | 66 | No | No | No | No | 1300 | Yes | No |
| 38 | C | M | 56 | No | No | No | No | 2831 | Yes | No |
| 39 | C | M | 56 | Yes | No | Yes | No | 6194 | Yes | No |
| 40 | C | M | 64 | No | No | No | No | 1801 | Yes | No |
| 41 | C | F | 77 | No | No | No | Yes | 1670 | Yes | No |
| 42 | C | F | 72 | No | No | No | No | 1623 | Yes | No |

Supplementary Table1 **Detailed clinical data of all patients**
